# Combatting negative bias: a mental contrasting and implementation intentions online intervention to increase help-seeking among individuals with elevated depressive symptomatology

**DOI:** 10.3389/fpsyg.2023.1145969

**Published:** 2023-06-15

**Authors:** Amanda R. Keeler, Liesl A. Nydegger, William D. Crano

**Affiliations:** ^1^Penn State Primary Care Research Laboratory, Department of Family and Community Medicine, Penn State College of Medicine, Hershey, PA, United States; ^2^Depression and Persuasion Research Laboratory, School of Social Science, Policy and Evaluation, Claremont Graduate University, Claremont, CA, United States; ^3^Mood Disorder Research Lab, Department of Psychiatry and Behavioral Health, Penn State College of Medicine, Hershey, PA, United States; ^4^Department of Health, Behavior and Society, Johns Hopkins University, Baltimore, MD, United States; ^5^Department of Kinesiology & Health Education, The University of Texas at Austin, Austin, TX, United States; ^6^Institute of Health Psychology and Prevention Science, School of Social Science, Policy and Evaluation, Claremont Graduate University, Claremont, CA, United States

**Keywords:** depression, implementation intentions, mental contrasting, negative bias, help-seeking, online intervention, MCII

## Abstract

**Background:**

There are many reasons why individuals with depression may not seek help. Among those with elevated depressive symptomatology, some previous interventions aimed at increasing help-seeking have unintentionally decreased help-seeking intentions. Beck’s cognitive theory of depression posits that individuals with elevated depressive symptomatology process information differently from those without depression (i.e., increased cognitive errors, negative bias); potentially explaining the iatrogenic results of previous interventions. Mental contrasting and implementation intentions (MCII; a self-regulatory strategy) interventions have successfully influenced physical and mental health behaviors. However, MCII has not been used specifically for initiating help-seeking for depression. The goal of this research was to ascertain whether an online MCII intervention could increase *actual* help-seeking or the *intention* to seek help for depression.

**Method:**

Two online randomized pre-post experiments were conducted to measure the primary outcome measures 2 weeks post-intervention (Study 1 collected Summer 2019: information-only control [“C”], help-seeking MCII intervention [“HS”], and comparison MCII intervention [“E”]; Study 2 collected Winter 2020: “C” and “HS”). At Time 1, adults recruited from MTurk had a minimum Beck Depression Inventory (BDI-II) score of 14 (mild depressive symptoms) and were not seeking professional help.

**Results:**

Study 1 (*N* = 74) indicated that the intervention was feasible, provided preliminary support, and clarified intervention components for Study 2. Study 2 (*N* = 224) indicated that the HS group reported greater *intentions* to seek help and *actual* help-seeking than the C group. Proportionally, *actual* help-seeking was more likely among individuals who received the HS intervention and either did not *perceive* themselves as depressed at Time 2 or had BDI-II scores indicating that their depressive symptomatology decreased from Time 1.

**Limitations:**

Participation was limited to US residents who self-reported data.

**Discussion:**

These studies indicate that a brief online MCII intervention to encourage help-seeking is feasible and preliminarily successful. Future studies should consider using ecological momentary assessment measurements to establish the temporal precedence of intervention effects and whether MCII is effective for encouraging help-seeking among individuals prone to experiencing cognitive errors who may not be experiencing negative bias (e.g., bipolar disorder or anxiety). Clinicians may find this method successful in encouraging ongoing treatment engagement.

## 1. Introduction

Although depression is a serious condition that affects millions worldwide (James et al., 2018) and is a leading risk factor for suicide (World Health Organization, 2021); with help, depression can be effectively treated (Linde et al., 2015). However, many who experience symptoms of depression do not seek treatment (Mekonen et al., 2022): a recent meta-analysis indicated that spontaneous remission rarely occurs (Mekonen et al., 2022). The goal of the current studies was to investigate the utility of a brief, theory-based online intervention designed to increase help-seeking (interpersonal or professional) initiation for those with elevated depressive symptomatology.

There are many reasons why an individual with depression may not seek help, such as not knowing how to seek help (e.g., Henderson et al., 2013; Keeler and Siegel, 2016), general lack of knowledge about depression (e.g., Rüsch et al., 2011), fear of stigma (Corrigan, 2004; e.g., Henderson et al., 2013), or structural barriers (e.g., Carbonell et al., 2020). Interventions can address these issues successfully, inducing individuals to seek help, potentially even saving lives (e.g., Siegel et al., 2015; Parikh et al., 2018). However, some interventions aimed at increasing help-seeking for depression (or addressing common barriers to help-seeking) have indicated iatrogenic results (for examples see Christensen et al., 2006; Klimes-Dougan and Lee, 2010; Sin et al., 2011; Klimes-Dougan et al., 2013; Lienemann et al., 2013; Keeler and Siegel, 2016). This is particularly problematic when an intervention appeared to have been successful for individuals who were not depressed (e.g., Keeler and Siegel, 2016) and indicates the necessity to consider how individuals with depression may respond to interventions differently from general populations (Siegel et al., 2017). Beck’s (Beck, 1964; Beck, 1967; Rush and Beck, 1978; Beck, 1987; Beck and Alford, 2009; Beck and Bredemeier, 2016) cognitive theory of depression (CTD) helps to explain why this phenomenon may occur.

Beck’s CTD (see Clark and Beck, 2010) describes how elevated depressive symptomatology can alter how an individual may process information differently from an individual who is not depressed. Beck reasoned this is due to the depressogenic schema that involves faulty patterns in attitudes and cognitions (negative bias) leading to cognitive errors. The negative bias that leads to the negative triad of thinking negatively about themselves, the world, and the future, may amplify the perception of barriers to seeking care [e.g., not knowing how to seek help (Henderson et al., 2013; Keeler and Siegel, 2016)]. Beck’s CTD illustrates the importance of choosing a sample comprised of individuals with elevated depressive symptomatology to test an intervention. Individuals without depression cannot be expected to think or experience the world in the same manner (Ingram et al., 2008). Further, Beck’s CTD suggests that individuals with elevated depressive symptomatology make cognitive errors indicating an intervention requiring a decreased cognitive load may be optimal (Bowie et al., 2017). However, understanding CTD alone does not solve the problem of the iatrogenic results.

Although Gollwitzer’s theory of implementation intentions (Gollwitzer, 1990; Gollwitzer, 1993; Gollwitzer and Bargh, 1996) and Oettingen and Gollwitzer’s (2010) addition of mental contrasting to implementation intentions (MCII) is relatively new, the literature suggests MCII may provide an optimal theoretical basis to overcome these barriers for the proposed studies. MCII is a meta-cognitive, self-regulatory strategy that can be employed to initiate behavior change (Duckworth et al., 2013). On their own, implementation intentions are concise action plans using “if-then” statements set in advance between a given situation and the planned goal, bridging the gap between setting a goal (intentions) and outlining the exact mechanisms of how one plans to achieve the goal when a critical cue occurs (see Gollwitzer and Brandstätter, 1997; Gollwitzer et al., 2005; Gollwitzer and Sheeran, 2006). In theory, once the implementation intention is set, when the critical cues are experienced at a later time, individuals will complete the action plan quickly and without conscious effort (Bayer et al., 2009). Mental contrasting requires a modification in the *process* of how the implementation intention is *formed*. It includes using imagery to help elaborate on the positive future of goal achievement as well as on the negative reality of what is required to attain the positive future goal (Oettingen and Gollwitzer, 2010; Oettingen and Gollwitzer, 2018). Therefore, the goal of mental contrasting in implementation intentions is to motivate and prepare individuals cognitively to form and engage in implementation intentions realistically to achieve their personalized goal (Oettingen and Gollwitzer, 2010; Oettingen and Reininger, 2016). In practice, MCII has also recently been recently rereferred to as a WOOP strategy (wish [i.e., goal], outcome [i.e., positive future of goal] obstacle [i.e., elaboration on negative reality or barrier], plan [i.e., implementation intention]) when used in interventions (e.g., Oettingen and Reininger, 2016; Gollwitzer et al., 2018; Mutter et al., 2020; Monin et al., 2021).

Although MCII has effectively encouraged a wide range of goal achievement including physical health behaviors with moderate success (see Wang G. et al., 2021), its use among specific populations with mental health concerns is comparatively sparse (Toli et al., 2016). Of the existing literature in the field of mental health, MCII has shown initial promise with increasing specific goal attainment in samples of individuals with ADHD (e.g., Gawrilow et al., 2012), depression (Fritzsche et al., 2016), and schizophrenia (e.g., Sailer et al., 2015). Additional implementation intentions studies focused on general mental health populations including reducing self-harm (Armitage et al., 2016). Using MCII techniques may compensate for any personality features (e.g., perfectionism) that can otherwise lessen the effectiveness of forming implementation intentions alone due to mental contrasting’s ability to enhance the strength of commitment to forming and following through with implementation intentions (Oettingen and Gollwitzer, 2010). The focus on remaining realistic about the barriers to goal achievement and finding ways to overcome them may be especially useful for encouraging individuals with mental health concerns to achieve their personal goals (Fritzsche et al., 2016). This is particularly true for those experiencing the negative bias and cognitive errors associated with depression that can hinder the ability to process positive thoughts without acknowledging the current pervasive negative thoughts difficult (Beck, 1987; Clark and Beck, 2010). Despite calls for increased use of MCII interventions among individuals with mental health concerns (see Gollwitzer and Sheeran, 2006; Toli et al., 2016) and a theoretical model that appears well suited for use with depression, MCII remains underutilized in the current literature.

Another gap in the field is whether MCII can help encourage individuals experiencing troubling mental health symptoms to *initiate* help-seeking. The current mental health implementation intention literature is nearly devoid of help-seeking interventions, with only one study that focused on helping individuals *follow through* with a previously scheduled mental health appointment (i.e., Sheeran et al., 2007). Given that MCII inductions have been useful to help create a strong commitment to initiate other physical health behaviors (e.g., Christiansen et al., 2010), it seems plausible that using MCII to initiate help-seeking for mental health concerns may be a valid way to overcome the personality boundary conditions and ensure the individual forms sufficiently strong links between the critical cues and responses (Gollwitzer and Sheeran, 2006) through the more intense inductions used with MCII.

Based on the rationale that MCII shows promise to overcome the negative bias as proposed by CTD and that the personalized approach can be tailored to address the multitude of help-seeking barriers, this set of studies was designed to test a novel online intervention. The overarching goal was to increase mental health help-seeking initiation intentions and behaviors among individuals with elevated depressive symptomatology. A subset of analyses from a larger set of studies collected as part of a dissertation (Keeler, 2021), the focus of the current publication is narrowed in scope to focus exclusively on testing the efficacy of a newly designed online MCII intervention across 2 studies.

The primary goal of Study 1 was to test the feasibility of a novel online MCII help-seeking intervention to help pinpoint components that would be necessary to include in Study 2. The first study included both a comparison group that completed an MCII for an exercise goal and an information-only control group to ascertain if any group differences could result from either the length of the intervention or if completing any MCII intervention would be effective in increasing help-seeking for depression. The second study focused on testing the effects of the intervention with a larger sample once the online intervention was optimized based on Study 1 findings.

## 2. Study 1

There were several specific goals for the preliminary study including: (a) establishing the initial feasibility of implementing a pre-post online MCII intervention; (b) clarifying components to optimize the help-seeking MCII intervention for Study 2 including the need to use both a comparison and a control condition; and (c) testing the two hypotheses using three conditions (information-only control [“C”], help-seeking MCII intervention [“HS”], and comparison exercise MCII intervention [“E”]).

The first hypothesis focused on terminal help-seeking. The second examined if the intervention could influence help-seeking intentions, which could indicate a willingness to seek help in the future. H1: Participants in the HS group (i.e., those who received the HS MCII) intervention would be more likely to report initiation of help-seeking for depression during the intervention period than those who were in either the E MCII or the C conditions. H2: Participants in the HS group would be more likely to report greater *intentions to seek help* than those in the E MCII and the C conditions *regardless of whether they actually sought help* during the intervention.

### 2.1. Method

#### 2.1.1. Participants

Participants were recruited from Amazon’s Mechanical Turk platform (MTurk; Amazon Web Services, RRID: SCR_012854) with data collected from June – August 2019. All potential participants completed an IRB committee-approved online informed consent attesting to general inclusion criteria (18 or older, United States Resident, English fluent) before prescreening for full inclusion criteria in accordance with Siegel and Navarro’s (2019) recommendations to prescreen MTurk populations. Individuals were prescreened for elevated depressive symptomatology (having a minimum score of 14 on the BDI-II) and current help-seeking practices for depression (not currently seeking professional help) before being immediately invited to participate in the intervention.

A G*Power (RRID: SCR_013726) analysis for a between-within repeated measures ANOVA with interactions using three groups and two time points (i.e., *f* = 0.15, *α* = 0.05, 1-β = 0.95, *r* = 0.80 between measurements) indicated a total sample size of 72 would be required for adequate power to detect a moderately small effect. However, to account for a 20% dropout (approximately 5 participants per condition), the initial aim was 30 participants for each condition (MCII HS, E, and C). The plan to recruit 90 individuals with elevated depressive symptomatology was comparable to previously successful MCII interventions’ total sample sizes that included individuals with depression (e.g., *N* = 47, Fritzsche et al., 2016; *N* = 36, Sailer et al., 2015). The 2 week duration of the intervention was based on the lengths of previous MCII mental health interventions have ranged from two (Gawrilow et al., 2012) to 4 weeks (Sailer et al., 2015); the decision to err on a shorter follow-up period was due to the online nature of the study to minimize attrition.

#### 2.1.2. Design

**Figure 1:**
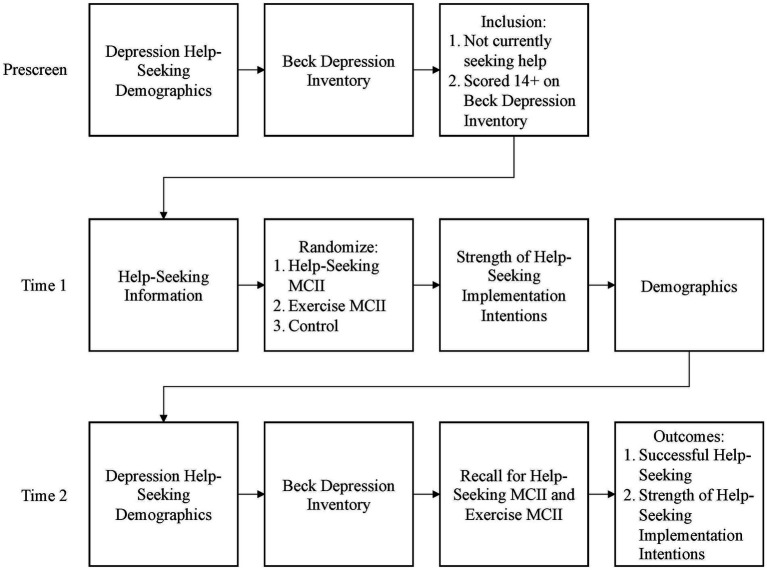
Study 1 layout. Time 2 occurred 2 weeks post intervention.

The study included three steps: prescreening, baseline (Time 1 [T1]), and termination (Time 2 [T2]) surveys (see Figure 1).

##### 2.1.2.1. Prescreen

All potential participants were invited to join a new longitudinal study testing a new goal achievement strategy. Those who chose to click the link were directed to affirm their provision of consent. Consenting participants were then asked to complete the BDI-II and a battery of help-seeking questions to determine eligibility. Those with a BDI-II score of 14 or above (indicating mild depressive symptomatology) and indicated that they had not sought help for their current bout of depression from a professional were immediately invited to participate in the main longitudinal study. Those who did not qualify or chose not to continue were provided with help-seeking information and paid $0.15 for their time.

##### 2.1.2.2. T1

Participants who agreed to continue the study read about the signs and symptoms of depression and completed several measures unrelated to the current analyses. After completing the scales, all participants were provided with information about the benefits of help-seeking for depression as well as a variety of low-cost and free help-seeking resources (see Supplementary material). All participants were asked to save this information as a memory aid in case the need to seek help arose. At this point, participants were randomly assigned to one of the three groups: (a) HS MCII intervention, (b) E comparison MCII, or (c) information-only (C). The HS and E groups each completed an MCII intervention ending with a personalized implementation intention. The HS group completed a personalized implementation intention to seek help for depression should the need arise. The E comparison group received similar information and resources regarding increasing exercise before completing a personalized implementation intention to increase their exercise by 20 min a week. The HS and E groups were directed to write down or print a copy of their implementation intention to keep as a reminder. All groups were asked to complete two versions of the Strength of Implementation Intention Scale (SIIS) to assess strength of implementation intentions to increase exercise and one for help-seeking before proceeding to the demographic questionnaire. All participants were thanked, compensated $1.50 for their time, and notified that they would be contacted in 2 weeks to complete the follow-up.

##### 2.1.2.3. T2

Two weeks later, all participants who consented to follow-up were notified via MTurk to complete the online battery of surveys in addition to a question to ask if they had sought help for depression during the previous 2 weeks. Both the HS and E groups were asked to reiterate the implementation intentions they established at T1 based on their reminder (HS group participants were prompted about help-seeking and the comparison E group was asked about exercise). All participants were directed to complete the help-seeking and exercise SIIS measures, asked if they had sought help for depression, and asked if they had increased their physical activity by 20 min during the previous 2 weeks. All participants were debriefed (including a tutorial on how to use the MCII technique) and paid $2.00.

#### 2.1.3. Materials

##### 2.1.3.1. Beck Depression Inventory-II

The BDI-II (Beck et al., 1996) is one of the most used depression assessments. The scale includes 21 groups of questions to determine levels of depressive symptomatology over the past 2 weeks. A composite score was calculated by summing the score of each question. Composite scores can range from 0 to 63 with higher scores indicating greater depressive symptomatology. Scores of 0–13 represent no to minimal, 14–19 mild, 20–28 moderate, and 29–63 severe depressive symptomatology (Beck et al., 1996).

##### 2.1.3.2. Depression history and demographics

A series of items were used to assess participants’ depression history in the screening survey. Items included: previous diagnosis of depression, current diagnosis of depression, current professional treatment for depression, and history of help-seeking from close others and mental health professionals. The T1 Survey ended with demographic items. The general items included characteristics such as gender, age, race, and insurance status that includes mental health coverage.

##### 2.1.3.3. Mental contrasting and implementation intentions related measures

Both the HS (intervention) and the E (comparison) groups completed an MCII exercise. The HS group focused on help-seeking for depression should the need arise and the E group focused on increasing exercise by 20 min a day. Additionally, all participants completed quantitative measures of intentions to seek help for depression.

###### 2.1.3.3.1. Induction

Two-thirds of the participants were randomly assigned to complete an MCII intervention (either help-seeking intervention [i.e., HS group] or physical activity comparison [i.e., E group]). For participants randomly assigned to complete either the HS or E MCII, this study used a multistep process implementation intention induction modeled after Sailer et al. (2015) aimed at increasing exercise for individuals with schizophrenia. All participants started by reading a short informational text that indicated behavior change was desirable, feasible, and how obstacles could be overcome. Afterward, participants were led through the following writing prompts:

- Participants identified a specific goal (the HS group were asked to pick a goal related to depression help-seeking; the E group were asked to choose a goal to increase exercise by 20 min per day).
- Asked to take a moment to imagine and write down the positive future of achieving their goal and to list four positive outcomes related to achieving goal (e.g., feeling happier or healthier).
- Mentally contrast the positive future with the current barrier or obstacle to achieving goal by listing four barriers (e.g., too tired, scared).
- Think about their biggest barrier or obstacle and write down ways to overcome it (e.g., enlist a friend’s help).
- Formulate an implementation intention plan to overcome the barrier in the form of an “if-then” plan.

This plan was copied three times in accordance to Sailer et al.’s methodology (p. 5). Similar to Fritzsche et al. (2016), participants were asked to screenshot or write down their implementation intention to act as a reminder.

###### 2.1.3.3.2. Follow-up questions

Inspired by Fleig et al. (2017), two questions assessed participants’ perceptions of both the viability (i.e., does the participant have the resources to carry out their implementation intention plan) and instrumentality (i.e., belief their action plan can help them achieve the goal of seeking help if needed) of the implementation intention to seek help they created, which was proposed to influence the likelihood of implementation intentions leading to the enactment of goal-directed behavior. Both questions were asked at T1 and T2 and were rated on a 7-point Likert scale ranging from 1 (“Strongly Disagree”) to 7 (“Strongly Agree”).

###### 2.1.3.3.3. Strength of implementation intentions scales

To quantitatively measure implementation intentions, we used a modified version of Nydegger’s Strength of Implementation Intentions Scale (SIIS; Nydegger et al., 2013, 2017). The scale was developed to assess the perceived strength of the link between the critical cue (e.g., when, where, and specific emotional trigger) and the precise action the individual is planning in response in order to achieve their goal (Nydegger et al.,2017). The SIIS has demonstrated acceptable internal consistency reliability (*α* = 0.96) with a focus on condom use and it was originally written so that it could be modified to fit different study and sample requirements by changing the target wording to be appropriate to various goals. The questions for these studies were modified with Dr. Nydegger’s guidance for the goals of help-seeking (SIIS HS; 7 questions) and increasing exercise (SIIS E; 5 questions) with questions rated on a 6-point Likert scale ranging from 1 (“Strongly Disagree”) to 6 (“Strongly Agree”).

###### 2.1.3.3.4. Success of MCII

At T2, all participants were asked to rate their perceived level of success related to increasing their exercise and help-seeking behaviors. Both questions were rated on a 6-point Likert scale ranging from 1 (“Strongly Disagree”) to 6 (“Strongly Agree”) with a “Not Applicable” option.

###### 2.1.3.3.5. Attention checks

In addition to the prescreening and VPN/geolocation, these studies utilized both quantitative and qualitative attention checks to prevent noted issues with fraudulent MTurk data (Kennedy et al., 2020). Three scale-embedded, quantitative attention checks directed participants to select specific response options as a measure of attention. Additionally, individuals in the E and HS groups had their MCII answers examined for whether the topic of MCII’s were on target, if directions were followed, and at T2, whether the participant remembered their MCII. All participants regardless of the data analysis plan (modified intention-to-treat or per-protocol) were required to pass the simple quantitative attention checks for quality control.

#### 2.1.4. Data cleaning and analysis plan

The pre-established data analysis plan required that the hypotheses would be analyzed in two ways: modified intention-to-treat (ITT) and per-protocol (PP). The rationale for using both is that ITT provides a more conservative estimate of the effectiveness of the intervention and is preferred by the Federal Food and Drug Administration for randomized control trials (see Day, 2008; Gupta, 2011). ITT analyses include all participants who had been randomized regardless of whether they dropped out of the study. This study pre-established the modification that participants would need to pass all quantitative attention checks (see Day, 2008) for inclusion for quality control due to the online nature of the study (Kennedy et al., 2020).

For the PP analyses, participants were excluded if they dropped out of the intervention before completion or missed any of the attention checks. Additionally, for the PP analyses, the content of the MCII was examined for those in the HS and E groups to explore whether the participants appeared to take the exercise seriously (e.g., were they on topic?) and at follow-up, did the participants indicate they remembered the general theme of their MCII? When the results of the ITT and PP analyses were the same, only the ITT results are reported. Multivariate outliers were removed based on Mahalanobis distance and univariate outliers based on Cook’s distance. The intervention and information-only control groups’ data were compared using non-parametric measures to ensure equivalence at T1.

### 2.2. Study 1 results

#### 2.2.1. Data cleaning

Data were analyzed with SPSS 27 software (IBMCorp, Released 2020; RRID:SCR_019096) in two ways: ITT and PP according to the established data plan.

##### 2.2.1.1. ITT

Of the total individuals (*N* = 981) who responded to the MTurk post, *n* = 117 did not consent, *n* = 461 had BDI-II scores lower than 14, *n* = 85 of individuals with BDI-II scores 14 and above but sought help for depression, and *n* = 179 participants did not pass one or both attention checks. The total number of participants per ITT at T1 was *N* = 139 with *n* = 38 HS, *n* = 39 E, and *n* = 62 C agreeing to be contacted for follow-up. Due to attrition of 55 between T1 and T2, the total possible number of participants for whom follow-up was possible was 83 (BDI-II T1 = 25.92 ± 9.59 (min 14, max 59), BDI-II T2 = 23.80 ± 10.59 (min 6, max 53), 36 ± 11.7 years, 57% Female, 77% white) with *n* = 19 HS, *n* = 23 E, and *n* = 41 C.

##### 2.2.1.2. PP

To establish the PP sample, the final T1 ITT sample was used as the initial starting point (*N* = 139). When examining the data to establish the PP sample, 55 participants were lost to attrition, eight participants were excluded based on blatantly not taking the MCII exercise seriously (e.g., “If I win the lottery, then I guess I’ll exercise more”) or showing no indication that they remembered forming an MCII (e.g., “I do not recall” or “I do not have a copy”). Two univariate outliers were identified using Cook’s Distance; no multivariate outliers were found via Mahalanobis distance. The final PP total was *N* = 74 [HS = 17, *E* = 17, *C* = 40, BDI-II T1 = 24.74 ± 9.32 (min 14, max 59), BDI-II T2 = 23.54 ± 10.26 (min 6, max 53), 35.4 ± 11.8 years, 57% Female, 76% white].

The HS and C groups’ data were compared to ensure equivalence at T1. Kruskal-Wallis for independent samples tests were used to assess group independence to determine any significant differences between groups for age, and chi-square tests were used to test for group differences in gender; no significant differences were observed (PP or ITT). See Table 1 for the full demographics and Table 2 depression help-seeking demographics for both the PP and ITT samples. Table 3 reports the descriptive information, and reliability information for the measures obtained in Study 1.

**Table 1:** Study 1 sample demographics.

Age *M* (*SD*)	PP *n* (%)	ITT Time 1 *n* (%)	ITT Time 2 *n* (%)
	35.4 (11.8)	35.7 (11.7)	36 (11.7)
Group assignment			
Help-seeking	17 (23.0)	38 (27.3)	19 (22.9)
Exercise	17 (23.0)	39 (28.1)	23 (27.7)
Control	40 (54.0)	62 (44.6)	41 (49.4)
Gender			
Male	31 (41.9)	56 (40.3)	34 (41.0)
Female	42 (56.8)	81 (58.3)	47 (56.6)
Prefer not to say	1 (1.4)	2 (1.4)	2 (2.4)
Ethnicity/Race			
African American/Black	5 (6.8)	15 (10.8)	5 (6.0)
Asian	7 (9.5)	13 (9.4)	7 (8.4)
Hispanic/Latinx	3 (4.1)	5 (3.6)	3 (3.6)
White	56 (75.7)	100 (71.9)	64 (77.1)
Other	0 (0)	2 (1.4)	1 (1.2)
Highest level of education			
Some high school	0 (0)	1 (0.7)	0 (0)
Graduated high school	11 (14.9)	17 (12.2)	13 (15.7)
Some college	18 (24.3)	40 (28.8)	20 (24.1)
Associate degree	5 (6.8)	11 (7.9)	5 (6.0)
Bachelor’s degree	34 (45.9)	62 (44.6)	38 (45.8)
Master’s degree or higher	6 (8.1)	8 (5.8)	7 (8.4)
Marital status			
Single	36 (48.6)	63 (45.3)	38 (45.8)
Married/committed relationship	30 (40.5)	55 (39.6)	34 (41)
Divorced	6 (8.1)	17 (12.2)	8 (9.6)
Other	0 (0)	1 (0.7)	0 (0)
Prefer not to say	2 (2.7)	3 (2.2)	3 (3.6)
Insurance includes ANY mental health			
No	20 (27.0)	43 (30.9)	22 (26.5)
Yes	42 (55.4)	74 (53.2)	46 (55.4)
I do not know	13 (17.6)	22 (15.8)	15 (18.1)

ITT, Modified intention-to-treat; PP, Per Protocol. PP *n* = 74, ITT T1 *n* = 139, ITT T2 *n* = 83.

**Table 2:** Study 1 depression help-seeking demographics.

	PP *n* (%)	ITT T1 *n* (%)	ITT T2 *n* (%)
Have you ever believed you were depressed but did not seek help?			
No	15 (20.3)	30 (21.6)	16 (19.3)
Yes	59 (79.7)	109 (78.4)	67 (80.7)
Have you ever sought help for depression from a loved one?			
No	42 (56.8)	75 (54)	48.8 (57.8)
Yes	32 (43.2)	64 (46)	35 (42.2)
Have you ever sought help for depression from a professional?			
No	42 (56.8)	76 (54.7)	45 (54.2)
Yes	32 (43.2)	63 (45.3)	38 (45.8)
Do you believe you currently have depression?			
No	26 (35.1)	49 (35.3)	30 (36.1)
Yes	48 (64.9)	90 (64.7)	53 (63.9)
Are you currently diagnosed with depression?			
No	60 (81.1)	111 (79.9)	66 (79.5)
Yes	14 (18.9)	28 (20.1)	17 (20.5)
Have you ever been diagnosed with depression?			
No	46 (62.2)	85 (61.2)	49 (59.0)
Yes	28 (37.8)	54 (38.8)	34 (41.0)
Are you currently seeking professional help for depression?			
No	74 (100)	139 (100)	83 (100)
Yes	0 (0)	0 (0)	0 (0)

ITT, Modified intention-to-treat; PP, Per Protocol. T1, Time 1; T2, Time 2. PP *n* = 74, ITT T1 *n* = 139, ITT T2 *n* = 83.

**Table 3:** Study 1 measure means, standard deviations, and internal consistency (if applicable).

Measure	N items	PP T1		PP T2		ITT T1		ITT T2	
		*M* (*SD*)	*α*	*M* (*SD*)	*α*	*M* (*SD*)	*α*	*M* (*SD*)	*α*
BDI	21	24.74 (9.32)	0.87	23.54 (10.26)	0.90	25.92 (9.59)	0.88	23.80 (10.59)	0.90
SIIS SH	–	26.64 (7.89)	–	26.47 (7.36)	–	26.62 (8.35)	–	25.98 (7.96)	–
HS	7	30.71 (5.26)	0.88	29.76 (5.89)	0.85	30.26 (6.62)	0.91	29.21 (8.22)	0.93
EX	7	27.47 (8.00)	0.95	24.94 (7.44)	0.93	27.21 (9.05)	0.96	23.48 (7.80)	0.94
C	7	24.58 (8.20)	0.93	25.73 (7.60)	0.94	24.02 (8.09)	0.93	25.88 (7.57)	0.94
SIIS EX	–	19.84 (5.24)	–	19.92 (5.77)	–	19.80 (6.32)	–	19.34 (6.34)	–
HS	5	20.47 (5.57)	0.92	20.71 (6.81)	0.96	19.50 (6.95)	0.96	19.05 (8.11)	0.98
EX	5	18.68 (5.36)	0.77	21.06 (5.30)	0.90	22.00 (5.99)	0.90	19.70 (6.34)	0.94
C	5	18.68 (5.36)	0.91	19.10 (5.51)	0.90	18.60 (5.84)	0.92	19.27 (5.55)	0.90
MCII SH success	1	–	–	3.58 (1.73)	–	–	–	3.45 (1.71)	–
MCII EX success	1	–	–	3.53 (1.66)	–	–	–	3.59 (1.71)	–

Means, standard deviations, observed alpha (if applicable). T1, Time 1; T2, Time 2; BDI, Beck Depression II inventory scores; C, information only control; SIIS, Strength of Implementation Intentions Scale; EX, Exercise; SH, Seek Help; ITT, Intend to Treat; PP, Per Protocol; MCII, Mental Contrasting and Implementation Intentions Intervention.

#### 2.2.2. H1

**Figure 2:**
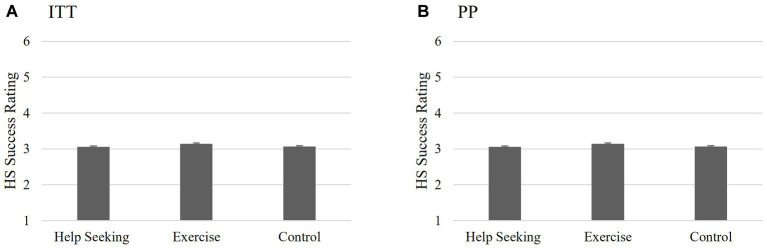
Study 1 Hypothesis 1: Success of help-seeking intervention. Non-significant interaction between groups on help-seeking success indicating that completing the HS MCII intervention had no significant effect on perceived help-seeking success 2 weeks post-intervention for either the ITT **(A)** or PP **(B)** analyses. Scores reported are the means for perceived success of help-seeking scores and the bars are the standard error.

The first hypothesis proposed that the HS group would be more likely to report that they *initiated* help-seeking for depression during the intervention period than those in either the comparison (E) or the control conditions (C). Individuals who responded with “Not Applicable” were treated as missing (ITT *n* = 8; PP *n* = 2) resulting in there being no differences between the ITT and PP participant samples and therefore, no differences in the analysis outcome. The one-way ANOVA analyses did not support this hypothesis *F*(2,74) = 0.046, *p* = 0.995; see Figure 2A for ITT and Figure 2B for PP. Individuals in the HS group were no more likely to report *actually* seeking help at T2 than those in the E or C groups.

#### 2.2.3. H2

Two-way mixed ANOVAs were used to test if there were group differences in *intentions to seek help* for depression from T1 to T2 *regardless* of whether participants *actually* sought help. H2 predicted that the HS group would report greater intentions to seek help from T1 to T2 for depression as measured by the SIIS HS than the E and C groups. GLM repeated measures function was used to conduct the 2 (SIIS HS: T1 and T2) X 3 (group: HS, E, C) ANOVA. Sphericity can be assumed since there were only two levels of the repeated measure. Bonferroni corrections were used for *post hoc* contrasts to account for multiple testing (Field, 2018). The results of this analysis varied depending on the sampling method.

**Figure 3:**
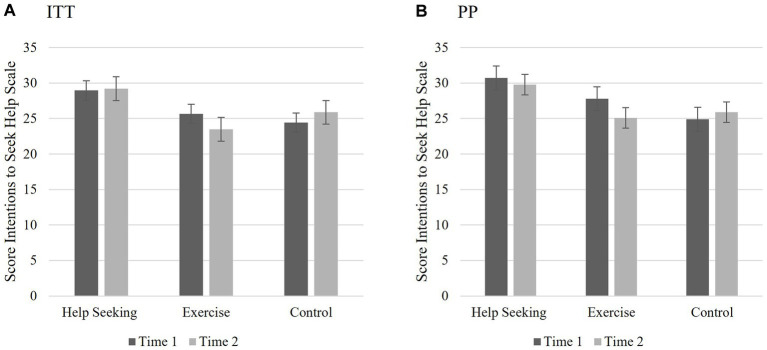
Study 1 Hypothesis 2: Changes to intentions to seek help. **(A)** ITT: Illustrating the non-significant interaction between groups and SIIS HS over time; completing the HS MCII intervention had no significant effect on intentions to seek help over time for the ITT analyses; However, **(B)** PP: indicated significant main effect for group and interaction between group and SIIS HS over time. Those who received HS intervention had greater intentions seek help across both time points compared to the other conditions. Scores reported are the means for the SIIS HS scores T1 and T2 and the bars are the standard error.

When examining the ITT sample, the analyses indicated that there were no significant main effects for scores on the SIIS HS over time (*F*(1,80) = 0.035, *p* = 0.853, partial *η*^2^ < 0.001) or group (*F*(2,80) = 2.361, *p* = 0.101, partial *η*^2^ = 0.06). Additionally, there was no significant interaction between group and scores on the SIIS HS over time (*F*(2,80) = 2.035, *p* = 0.137, partial *η*^2^ = 0.05). The results indicate that the hypothesis was not supported using the ITT sample; completing the HS MCII had no effect on SIIS scores over time. See Figure 3A for means and standard error of scores.

For the PP sample, the results indicate that completing the HS MCII influenced SIIS HS scores over time. Analyses revealed no main effect for the SIIS HS, *F*(1,73) = 1.067, *p* = 0.305, partial *η*^2^ = 0.01, indicating that individual’s scores on the SIIS HS measure did not vary significantly from T1 to T2. However, there was a main effect for group differences, *F*(2,73) = 3.364, *p* = 0.04, *η*^2^ = 0.08), and a significant interaction between group and scores on the SIIS HS over time (*F*(2,73) = 3.713, *p* = 0.029, partial *η*^2^ = 0.09). Examining the pairwise comparisons, there was a significant difference between the HS and C groups (*M*diff = 5.090, *p =* 0.035, 95% CI:(0.278; 9.090). There were no significant differences between the HS and the E groups, nor between the E and C groups. See Figure 3B for means and standard error of scores.

### 2.3. Study 1 discussion

Study 1 was designed as a preliminary study to accomplish three primary goals: (a) establish the feasibility of conducting an entirely online MCII intervention, (b) clarify areas of improvement for Study 2, and (c) test the primary intervention hypotheses. Despite high attrition, it was possible to translate an MCII intervention to work online for individuals with depression. During the intervention, there were clearly features of the study that required improvement. The following sections of the discussion briefly examine the results of the hypothesis tests and their implications for Study 2.

#### 2.3.1. Hypotheses

It was expected that individuals in the HS group would be more likely to seek help for their depressive symptoms (H1) and would be more likely to report a greater intention to seek help over time (H2) when compared to the E and C groups. Like all the group-based analyses, H1 and H2 may have suffered by the unequal and smaller proportion of participants in the HS (ITT *n* = 19, PP *n* = 17) and E (ITT *n* = 23, PP *n* = 17) groups compared to the C group (ITT *n* = 41, PP *n* = 40).^1^ Although there were no significant group differences in reported success in actual help-seeking for depression (H1), the hypothesized significant group differences and interaction was found for the PP sample in the Strength of Implementation Intentions Scale for Help-Seeking (SIIS HS). Since there were no significant differences between the E and C groups or the HS and E groups for H2, it seems reasonable to limit Study 2 to control and experimental groups. By limiting the number of groups, it will be possible to achieve greater statistical power in the forthcoming analyses.

One alternative explanation for the lack of intervention effects is whether participants in the HS group failed to achieve their goals due to a lack of perceived utility or a perceived lack of resources. Despite being conducted, an exploratory mixed ANOVA using the two questions inspired by Fleig et al. (2017), assessing instrumentality and resources was not included in the results section due to the highly speculative nature of the underpowered results (eight participants affirmed they achieved success at their goal and eight participants did not). Each of the questions were scored on a 1 (“Strongly Disagree”) to 7 (“Strongly Agree”) measure with average item scores of the non-successful individuals ranging from *M* = 4.38 (*SD* = 2.39) to *M* = 5.75 (*SD* = 1.28) and successful individuals were similar for both questions ranging from *M* = 5.63 (*SD* = 0.916) to *M* = 6.13 (*SD* = 0.84) indicating that individuals generally believed they saw utility in goal setting and had the resources to carry them out at both T1 and T2. However, despite the small number of participants, the analysis seemed to suggest that utility and resources did not make a significant difference in level of success, nor did it appear that the scores changed significantly over time. Although this analysis examining an alternative explanation was underpowered, and thus, the likelihood of a Type II error is high (Crano et al., 2014), the means of perceived utility and perceived resources all being rated positively indicate it was likely that ceiling effects occurred. Depending on the results of Study 2 these should be examined again with a larger sample to rule out the alternative explanation that perceived utility or resources may influence help-seeking outcomes.

Despite the ~40% attrition between T1 and T2 for the modified ITT MTurk sample, it was possible to obtain an adequate sample of individuals with elevated depressive symptomatology based on the power calculations. Other studies have also described issues with attrition in MTurk samples (Zhou and Fishbach, 2016; Hauser et al., 2018) and stressed the importance of tempering conclusions drawn with elevated attrition levels. Several modifications to the study design were implemented to decrease attrition in Study 2.

#### 2.3.2. Modifications for Study 2

Though the results of the hypotheses from Study 1 were weak, several key lessons were used to design Study 2. For example, the results of this preliminary study suggested answers to questions such as: (a) what level of attrition should be expected? (~40% without modifications), (b) is there evidence that new HS SIIS scale is reliable? (yes), and (c) did the data indicate the necessity for both a control and comparison group? (no). Although there was limited success with changing intentions to seek help in 2 weeks using the more liberal estimate of SIIS HS in the PP analysis, Zhou and Fishbach (2016) noted the importance of tempering the excitement due to the large percentage of attrition–especially considering the small sample size. Due to the high attrition noted in this study, the authors decided that a longer duration between Times 1 and 2 or adding a booster session between time points would only exacerbate attrition.

Planned changes to avoid low power in the second study included conducting new power analyses based on observed measure correlations from Study 1 rather than using estimates and oversampling with the expectation of attrition. Further, Study 2 could conserve power by focusing only on the two groups that illustrated significant differences: C and HS. To decrease the burden on participants, the questionnaires were shortened to only necessary measures to test for the intervention effects. After analyzing Study 1 data, it seemed plausible that an additional alternative explanation for intervention effects to seek help could be related to participants’ perceptions of their depression; prompting the question to be asked at both time points in Study 2.

## 3. Study 2

With the study modifications in place, Study 2 aimed to explore the two intervention-based hypotheses explored in Study 1: H1: Participants in the HS group (i.e., those who receive the HS MCII intervention) would be more likely to report that they initiated help-seeking for depression during the intervention period than those who were in C. H2: Participants in the HS group would be more likely to report greater *intentions to seek help* (as measured by the SIIS HS) than those in C *regardless of whether they actually sought help* during the intervention.

### 3.1. Method and materials

#### 3.1.1. Participants

Participants were again recruited from Amazon’s MTurk (RRID:SCR_012854) with data collected between February – early March 2020. To participate in the prescreening, participants were notified that they must be an English-speaking US resident and at least 18 years old. Again, to be consistent with Siegel and Navarro’s (2019) recommendation of prescreening MTurk populations rather than explicitly listing inclusion criteria for online surveys, the sample was prescreened for depressive symptomatology (having a minimum score of 14 on the BDI-II) and current help-seeking practices for depression (not currently seeking professional help) prior to being immediately invited to participate in the first part of the intervention. Individuals who participated in Study 1 were not eligible to participate. A G*power analysis for a between-within repeated measures ANOVA with interactions using two groups (HS and C), two measurements (*r* based on Study 1’s correlation between T1 and T2 SIIS HS; *f* = 0.15, *α* = 0.05, 1-β = 0.95, *r* = 0.65), indicated a total sample size of 104 (Faul et al., 2009). Due to the large proportion of participants in the first study who did not pass the attention checks (Study 1 = 56%) and the significant attrition from T1 to T2 (40%), screening continued until 345 participants passed the initial screening.

#### 3.1.2. Design

**Figure 4:**
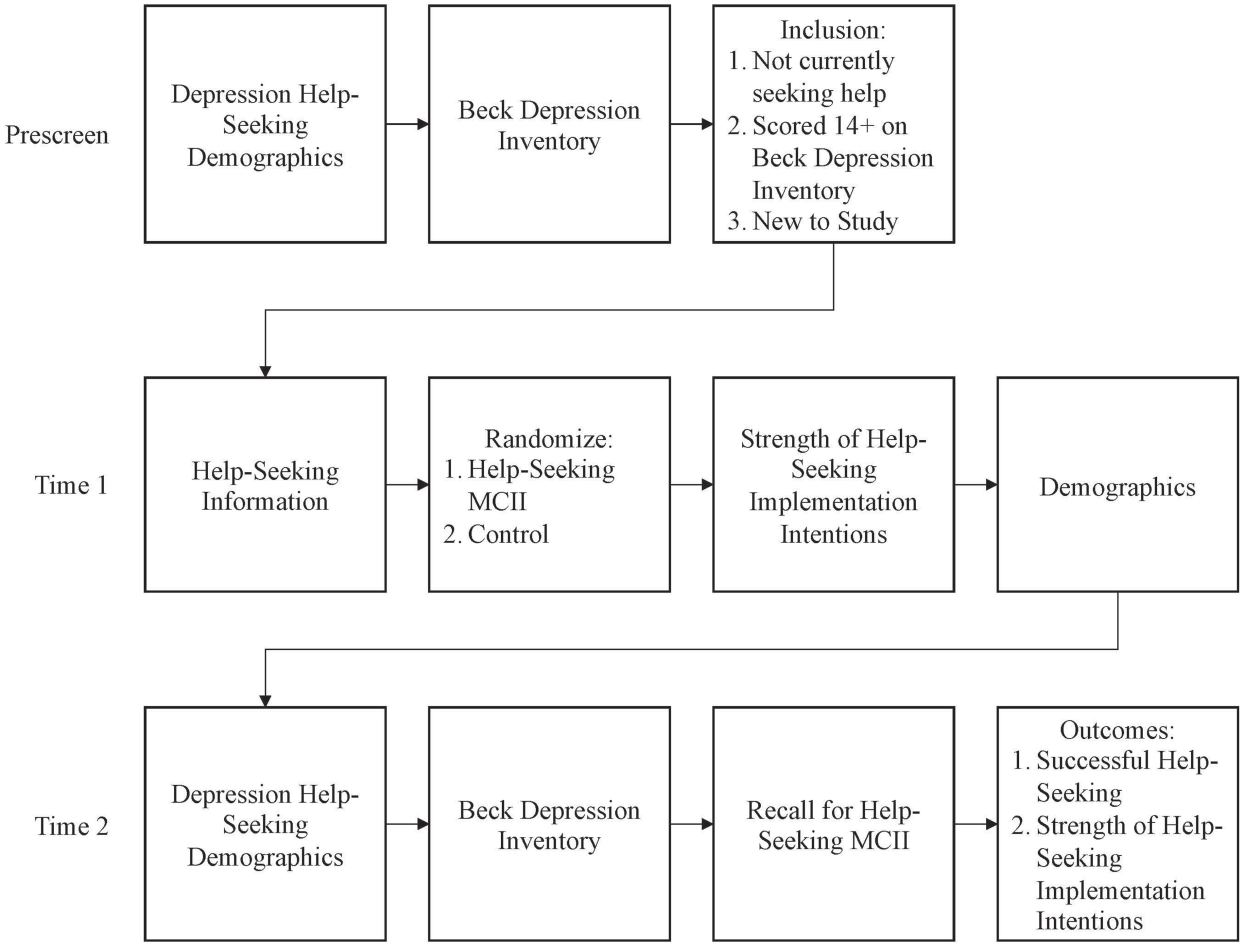
Study 2 layout. Time 2 occurred 2 weeks post intervention.

The design of Study 2 was a slightly modified version of Study 1. Study 2 used only the experimental (HS) and information-only control (C) groups and focused on the measures necessary for testing the intervention effects (i.e., removal of unrelated scales, additional depression demographics at T2). Otherwise, the design mirrored Study 1 (see Figure 4).

#### 3.1.3. Measures

To reduce time and cognitive fatigue, the measures were limited to what was needed to study the intervention effects. The measures included depressive symptomatology (i.e., BDI-II, depression demographics) and MCII measurements (i.e., induction, intervention success, MCII instrumentality, MCII viability, and SIIS HS). Full descriptions of the measures are in Study 1.

#### 3.1.4. Data cleaning and analysis plan

The data analysis plan mirrored Study 1 and included both the modified ITT and PP analyses. When both ITT and PP analyses are both significant, only the more conservative ITT results are provided in text.

### 3.2. Study 2 results

Of the 2,134 respondents to the MTurk post, 382 did not consent, and 1,005 had BDI-II scores lower than 14. Of the 712 who had BDI-II scores of 14 or above, 137 were excluded for responding that they previously took a similar survey and 206 were disqualified for currently seeking professional help. Of the 345 individuals who consented to continue with the study, an additional 24 were removed for missing the attention check, resulting in T1 ITT total *N* = 321 (HS = 149, C = 172). For the T2 ITT analyses, 44 were lost in the HS group (32 attrition, 12 failed attention check; *n* = 105) and 44 participants in the C condition were lost to attrition (4 additional for failed attention check; *n* = 124). Therefore, the total ITT sample analyses were conducted with 228 [HS = 105, C = 123, BDI-II T1 = 24.32 ± 9.07 (min 14, max 54), BDI-II T2 = 24.01 ± 10.62 (min 2, max 54), 37.2 ± 12.1 years, 66% Female, 67% white] participants with the total attrition from T1 to T2 reduced, compared to Study 1, to 24% and quality control removals were reduced to 5%.^2^

For the PP analyses, an additional 19 participants in the HS condition were excluded after reading through the MCII for either blatantly not taking the exercise seriously (e.g., copy and pasting the question in the answer box), or indicating no recollection of completing the exercise. One univariate outlier and four multivariate outliers were removed bringing the total for the PP sample to 205 [HS = 86, C = 119, BDI-II T1 = 24.44 ± 9.41 (min 14, max 54), BDI-II T2 = 23.93 ± 10.93 (min 2, max 54), 37.2 ± 12.4 years, 68% Female, 69% white]. See Table 4 for the full demographics and Table 5 depression help-seeking demographics.

**Table 4:** Study 2 sample demographics.

	PP *n* (%)	ITT Time 1 *n* (%)	ITT Time 2 *n* (%)
Age *M* (*SD*)	37.4 (12.4)	36.0 (11.8)	37.2 (12.1)
Group assignment			
Help-seeking	86 (42.0)	149 (46.4)	105 (45.9)
Control	119 (58.0)	172 (53.6)	123 (54.1)
Gender			
Male	66 (32.2)	122 (38.0)	76 (33.3)
Female	139 (67.8)	198 (61.7)	151 (66.3)
Prefer not to say	0 (0)	1 (0.3)	1 (0.4)
Ethnicity/Race			
African American/Black	17 (8.3)	31 (9.7)	19 (8.3)
Asian	21 (10.2)	33 (10.3)	26 (11.4)
Caucasian/White	142 (69.3)	213 (66.4)	153 (66.8)
Hispanic/Latinx	18 (8.8)	33 (10.3)	20 (8.7)
Multiethnic	5 (2.5)	8 (2.5)	7 (3.1)
Other	2 (1.0)	3 (0.9)	3 (1.2)
Highest level of education			
Some high school	0 (0)	2 (0.6)	0 (0)
Graduated high school	25 (12.2)	30 (9.3)	26 (11.4)
Some college	53 (25.9)	89 (27.7)	62 (27.2)
Associate degree	19 (9.3)	30 (9.3)	22 (9.6)
Bachelor’s degree	77 (37.6)	131 (40.8)	86 (37.7)
Master’s degree or higher	31 (15.1)	39 (12.1)	32 (14.0)
Marital status			
Single	72 (35.1)	119 (37.1)	79 (34.6)
Married/committed relationship	114 (55.6)	168 (52.3)	128 (56.1)
Divorced	15 (7.3)	27 (8.4)	16 (7.0)
Other	4 (2.0)	5 (1.6)	4 (1.8)
Prefer not to say	0 (0)	2 (0.6)	1 (0.4)
Insurance includes ANY mental health			
No	65 (31.7)	113 (35.2)	77 (33.8)
Yes	98 (47.8)	149 (46.4)	106 (46.5)
I do not know	42 (20.5)	59 (18.4)	45 (19.7)

PP *n* = 205, ITT Time 1 *n* = 321, ITT Time 2 *n* = 228.

**Table 5:** Study 2 depression help-seeking demographics.

	PP *n* (%)	ITT Time 1 *n* (%)	ITT Time 2 *n* (%)
Have you ever believed you were depressed but did not seek help?^a^			
No	53 (25.9)	90 (28.0)	61 (26.6)
Yes	152 (74.1)	231 (72.0)	167 (72.9)
Have you ever sought help for depression from a loved one?^a^			
No	94 (45.9)	161 (50.2)	112 (48.9)
Yes	111 (54.1)	160 (49.8)	116 (50.7)
Have you ever sought help for depression from a professional?^a^			
No	110 (53.7)	179 (55.8)	125 (54.6)
Yes	95 (46.3)	142 (44.2)	103 (45.0)
Do you believe you currently have depression?^a^			
No	85 (41.5)	138 (43.0)	96 (41.9)
Yes	120 (58.5)	183 (57.0)	132 (57.6)
Are you currently diagnosed with depression?^a^			
No	170 (82.9)	265 (82.6)	192 (83.8)
Yes	14 (17.1)	56 (17.4)	36 (15.7)
Have you ever been diagnosed with depression?^a^			
No	128 (62.4)	210 (65.4)	146 (63.8)
Yes	77 (37.6)	111 (35.6)	82 (35.8)
Are you currently seeking professional help for depression?^a^			
No	205 (100)	321 (100)	228 (100)
Yes	0 (0)	0 (0)	0 (0)
Have you sought help for depression from a loved one in the past 2 weeks?^b^			
No	135 (65.9)	–	155 (68.1)
Yes	70 (34.1)	–	73 (31.9)
Have you sought help for depression from a professional in the past 2 weeks?^b^			
No	183 (89.3)	–	204 (89.5)
Yes	22 (10.7)		24 (10.5)
Do you believe you currently have depression?^b^			
	82 (40)	–	92 (40.6)
	123 (0)	–	136 (59.4)

^a^Time 1 question; ^b^Time 2 depression question. ITT, Modified intention-to-treat; PP, Per Protocol. PP *n* = 74, ITT T1 *n* = 139, ITT T2 *n* = 83.

Kruskal-Wallis for independent samples was used to test for group independence to determine any significant differences between groups for age and Chi square tests were used to test for group differences in gender. No significant differences were observed (PP or ITT). Please see Tables 4, 5 for the demographics and reported depression help-seeking demographics for both the PP and ITT samples that were used for analyses. Scale analyses were completed for the study for both analysis methods (see Table 6).

**Table 6:** Study 2 measure means, standard deviations, and internal consistencies (if applicable).

Measure	N items	PP T1		PP T2		ITT T1		ITT T2	
		*M* (*SD*)	*α*	*M* (*SD*)	*α*	*M* (*SD*)	*α*	*M* (*SD*)	*α*
BDI	21	24.44 (9.41)	0.89	23.93 (10.48)	0.92	24.32 (9.07)	0.88	24.01 (10.62)	0.92
SIIS HS	–	26.64 (7.89)	–	26.47 (7.36)	–	26.83 (8.39)	–	25.90 (8.04)	–
HS	7	28.80 (7.16)	0.92	28.80 (7.16)	0.94	27.84 (7.37)	0.92	27.84 (7.37)	0.92
C	7	24.22 (8.36)	0.93	24.22 (8.36)	0.93	24.26 (8.25)	0.93	24.26 (8.25)	0.93
MCII HS success	1	–	–	3.19 (1.39)	–	–	–	3.21 (1.38)	–

Measures Means, standard deviations, observed alpha (if applicable). T1, Time 1; T2, Time 2; BDI, Beck Depression II inventory scores; SIIS, Strength of Implementation Intentions Scale; SH, Seek Help; ITT, Intend to Treat; PP, Per Protocol; MCII, Mental Contrasting and Implementation Intentions Intervention.

#### 3.2.1. H1

**Figure 5:**
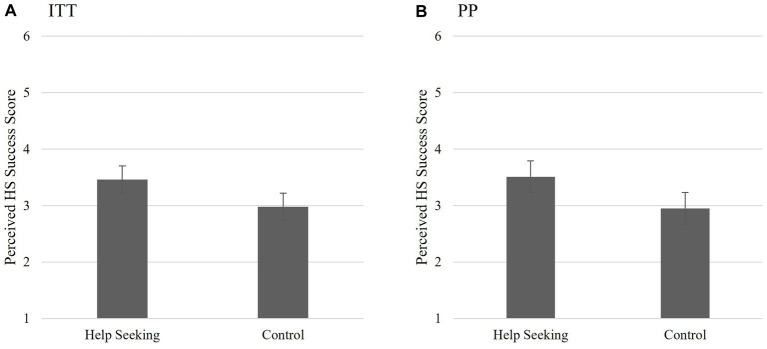
Study 2 Hypothesis 1: Success of help-seeking intervention. Those who received the intervention were more likely to report seeking help in the past 2 weeks than those who received information alone. This was supported for both the IIT **(A)** and **(B)** PP analyses Scores reported are the means for perceived success of help-seeking scores and the bars are the standard error.

H1 predicted that participants in the HS group would be more likely to report greater success in initiating help-seeking for depression during the intervention period than those in the C group. Individuals who answered this item as “Not Applicable” were excluded from this analysis (ITT *n* = 24, PP *n* = 23). The independent samples t-test analyses supported this hypothesis regardless of the sampling measure (*t*[202] = 2.509, *p* = 0.013, CI: 0.103, 0.860): Participants in the HS group were more likely to report greater mean help-seeking success at T2 than those in the C group. See Figure 5A (ITT) and Figure 5B (PP) for means and standard error of scores.

#### 3.2.2. H2

**Figure 6:**
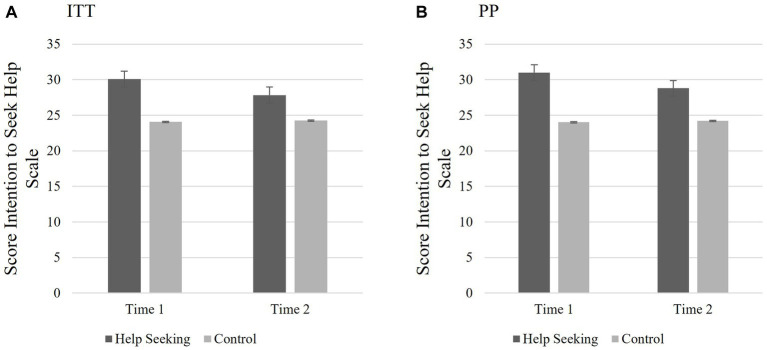
Study 2 Hypothesis 2: Changes to intentions to seek help. Illustrating the significant main effect for group and interaction between group and SIIS HS over time. Those who received HS intervention had greater intentions seek help across both time points compared to those who received information alone. This was supported for both the IIT **(A)** and PP **(B)** analyses. Scores reported are the means for the SIIS HS T1 and T2 and the bars are the standard error.

H2 predicted that participants in the HS group (i.e., those who completed the HS MCII) would be more likely to report greater *intentions to seek help* (as measured by the SIIS HS) than those in C *regardless of whether they actually sought help* during the intervention. Repeated measure mixed ANOVAs were used to test this hypothesis (SIIS HS scores T1, T2 x group). The results of this analysis supported this hypothesis regardless of testing the ITT or PP sample. The results found that completing the HS MCII positively influenced SIIS HS scores over time. Analyses revealed a statistically significant main effect for the SIIS HS, *F*(1,226) = 7.979, *p* = 0.005, partial *η*^2^ = 0.034, indicating that individual’s scores on the SIIS HS measure significantly varied from T1 to T2 for all participants. There was a main effect for group differences, (*F*(1,226) = 24.084, *p < 0*.001, *η*^2^ = 0.096). Examining the pairwise comparisons, there was a significant difference between the HS and C groups indicating that the HS groups scored significantly higher (*M*diff = 4.803, *p* < 0.001, 95% CI: 2.875; 6.732). There was a significant group x SIIS HS interaction, *F*(1,226) = 11.468, *p* = 0.001, partial *η*^2^ = 0.048. Although the HS group scored higher on the SIIS HS measure than the C group at both T1 and T2, the SIIS HS scores of the HS group decreased at T2. This decrease was not observed in the C group. See Figure 6A (ITT) and Figure 6B (PP) for means and standard error of scores.

#### 3.2.3. Exploratory Analysis 1: effect of perceived utility and perceived resources on successful help-seeking

Fleig et al. (2017) proposed that the success of MCII could be dependent on perceived utility and perceived resources. A mixed ANOVA tested a 2 (utility T1, utility T2) × 2 (resources T1, resources T2) x 2 (success/nonsuccess of intervention) for participants in the HS group. Individuals who rated their MCII success as 1–3 were considered unsuccessful (ITT *n* = 44, PP *n =* 35) and those who rated their MCII success as 4–6, (ITT *n* = 51, PP *n =* 42) were considered successful. Those who indicated that the question was not applicable (ITT *n* = 10, PP *n =* 9) were treated as missing. The goal was to explore the relationship among perceived success of their HS MCII, utility of the interventions, and the perceived availability of the resources to carry out the interventions.

**Figure 7:**
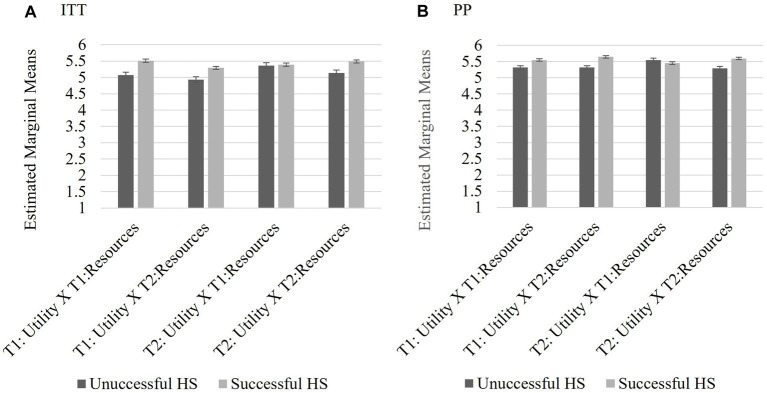
Study 2 Exploratory Analysis 1 3-way interaction: Utility x Resource x Success Ratings. For those who completed the HS MCII, there were no significant relationships between perceived utility, resources, nor interaction between utility and resources on perceived help-seeking success. Scores reported are the estimated marginal means for the interaction terms and the bars are the standard error for both the IIT **(A)** and PP **(B)** analyses.

The results of the analyses did not vary significantly based on analysis methodology, therefore the results reported in text are the from the ITT sample. There were no significant differences between scores on perceived utility, (*F*(1,93) = 3.054, *p* = 0.084, partial *η*^2^ = 0.032) or scores on perceived resources (*F*(1,93) = 1.066, *p* = 0.305, partial *η*^2^ = 0.011) between T1 and T2. Additionally, there was no significant differences between those who rated themselves as successful and those who did not, *F*(1,93) = 1.445, *p* = 0.232, partial *η*^2^ = 0.015. There were no interactions between perceived success and either perceived utility, *F*(1,93) = 1.622, *p* = 0.206, partial *η*^2^ = 0.017, nor perceived resources, *F*(1,93) = 0.278, *p* = 0.599, partial *η*^2^ = 0.003, over time. There were no significant interactions between perceived utility and perceived resources (*F*(1,93) = 0.560, *p* = 0.456, partial *η*^2^ = 0.006). Further, there was not a three-way interaction between perceived utility over time, perceived resources over time, and perceived success, *F*(1,93) = 1.848, *p* = 0.177, partial *η*^2^ = 0.019. Please see Figure 7A (ITT) and Figure 7B (PP) for estimated marginal means for the three-way interaction term and the standard error bars.

#### 3.2.4. Exploratory Analysis 2: effect of actual and perceived depression on successful help-seeking

Neither the perceived utility of goal setting nor having resources to carry out the MCII had a significant effect on perceived help-seeking success; thus, it seemed plausible that success was based on whether individuals believed help-seeking was needed. This alternative explanation was tested using both conditions by examining the potential role of perceived and actual depressive symptomatology in outcome ratings of perceived success for help-seeking.

The following analyses were conducted to determine if there were differences in frequencies in help-seeking success (dichotomous: 1 = success defined as reporting a score of 4 “slightly agree” to 6 “strongly agree” on the item relating to success of help-seeking; below 4 = 0) as a result of perceiving one was experiencing depression *regardless* of *actual depression* score (T2 “Are you currently depressed”; 1 = no, 0 = yes), actual depression score (BDI-II T2 dichotomous; 13 and below = 1 no to minimal; 14 and above = 0 at least mild symptomatology), and group (C; HS). The decision to dichotomize depression at a score indicating at least a mild level of depressive symptomatology (Hautzinger et al., 2009) was based on using the same cutoff in the screening measure. The rationale for dichotomizing and scoring all positive results (i.e., success, lower perceived depression, no-to-minimal BDI) in the same direction provided the clearest basis for understanding the participants’ perception of their success in achieving their goal of help-seeking and lowering depression. Individuals who indicated that the goal of increasing help-seeking for elevated depressive symptomatology was “not applicable” were excluded from these analyses (*n* = 24). For both sets of analyses, crosstab analyses were conducted ITT and PP as well as comparing the total sample to each of the conditions individually. If the results for the HS and C conditions indicated the same pattern, the total sample was utilized. MedCalc (RRID:SCR_015044) was used to calculate the odds ratios and confidence intervals. When both sample statistics were significant, only the ITT sample is listed in the text.

##### 3.2.4.1. Exploratory Analysis 2a: the effect of actual depression at T2 on successful help-seeking

The first chi-square analysis explored whether the categorical level of depressive symptomatology as measured by the BDI-II at T2 (no or minimal vs. mild or greater), influenced help-seeking success (successful vs. non-successful). The ITT and PP results followed the same pattern of results and indicated significant differences based on condition assigned at randomization. At least one cell had an expected count below 5 for the C group.

For the HS group, the pattern of results varied significantly based on whether the participant had a score of at least a mild level of depressive symptomatology at T2 on the BDI-II and perceived help-seeking success, ITT *X^2^*(1) = 7.969, *p* = 0.005 OR 11.825, CI: 1.460 to 95.790. Regardless of the sampling method, the odds ratio indicated the likelihood of reporting successful help-seeking was proportionally over 10 times greater for individuals whose depression scores fell to 13 or below on the BDI-II (no to minimal depression) than those continuing to score 14 and above indicating at least mild depression at T2.

**Figure 8:**
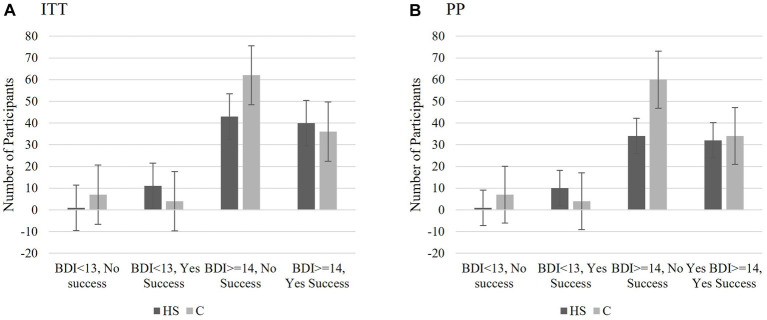
Study 2 Exploratory Analysis 2a: The effect of actual depression at T2 on successful help-seeking. Those who received the HS MCII and whose actual depression scores fell below the threshold for mild symptomatology at T2 (BDI-II = 0–13), were proportionally the most likely to report seeking help. Depression scores did not significantly influence the success in the control group. This was true for both the IIT **(A)** and PP **(B)** analyses. BDI scores 0–13 indicates no to minimal symptomatology; BDI scores 14 or higher indicate mild or greater symptomatology. The scores represent number of participants who fell into each categorical classification and the bars are the standard errors.

For the C group, a Bayes factor correction was used as a correction for the low expected cell count. For those in the control condition, the categorical level of depressive symptomatology did not affect whether a participant reported seeking help, ITT *X^2^*(1) = 0.001, *p* = 0.990, *BF* = 2.775, OR = 0.981, CI: 0.270 to 3.594. The Bayes factor indicated the probability of the data was only 0.36 times greater given the alternative hypothesis that BDI-II classification influences success rather than the null hypothesis for individuals in the control condition (ITT or PP). Please see Figure 8A (ITT) and Figure 8B (PP) for the number of participants in each classification and the standard error bars.

##### 3.2.4.2. Exploratory Analysis 2b: the effect of perceived depression at T2 on successful help-seeking at T2

The ITT and PP samples followed the same pattern of results when examining the influence of perceived depression (regardless of actual depressive symptomatology scores), and help-seeking success as EA2a. There was a significant relationship between perceived depression status and help-seeking success for individuals in the HS group, *ITT X^2^*(1) = 8.387, *p* < 0.001 OR 3.739, CI: 1.497 to 9.340; PP *X^2^*(1) = 3.902, *p* < 0.05, OR = 2.626, CI 0.995 to 6.929. The significant odds ratio for the ITT sample indicated that the probability of reporting success at help-seeking was 3.741 times higher for individuals in the HS group who did not perceive themselves as depressed at T2 than those who did. Despite a significant chi-square, the odds ratio for the PP fell short of reaching significance.

**Figure 9:**
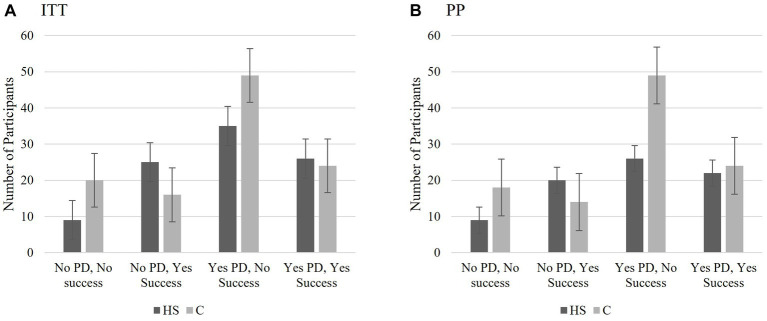
Study 2 Exploratory Analysis 2b: The effect of perceived depression at T2 on successful help-seeking at T2. **(A)** The significant odds ratio for the ITT sample indicated that the probability of reporting success at help-seeking was 3.741 times higher for individual in the HS group who did not perceive themselves as having elevated depressive symptomatology (PD) at T2 than those who did. **(B)** Despite a significant chi square, the confidence interval for the odds ratio was not significant for the PP sample for the HS group. There were no significant relationships between the PD at T2 and perceived success for the C group (ITT or PP). The scores represent number of participants who fell into each categorical classification and the bars are the standard errors.

However, for the control group, there was no significant difference between perceived depression status and help-seeking success regardless of sample, *ITT X^2^*(1) = 1.389, *p* = 0.292, OR = 1.633, CI: 0.720 to 3.705. Please see Figure 9A (ITT) and Figure 9B (PP) for the number of participants in each classification and the standard error bars.

### 3.3. Study 2 discussion

The goal of Study 2 was to test the MCII intervention using a streamlined set of procedures given the results of Study 1. Specifically, the hypotheses were designed to test whether an online MCII intervention could increase *actual* help-seeking or the *intentions* to seek help for depression. Despite discontinuing survey collection early due to the COVID-19 pandemic, attrition in the ITT sample was reduced to 24% with the modifications made from Study 1 (e.g., limiting the scales to the ones necessary for testing the intervention, updating the power analysis calculation, oversampling).

#### 3.3.1. Planned analyses

H1 indicated that the HS group was more likely to report seeking help at T2 than those in the control group. In addition, there was a significant interaction such that individuals in the HS group were more likely to report greater intentions to seek help for depression (as measured by SIIS HS) than those in the control condition over both time points; the individuals in the HS group had a dip in SIIS HS scores over time, which was not observed in the C group (H2). The literature is mixed regarding how long implementation intentions will last without a booster; Martin et al. (2011) found that a simple implementation intention intervention that involved similar implementation intention formation focusing on contraception reduced unplanned pregnancy and emergency contraception consultations among young women over the course of 2 years for the intervention group. Martin et al.’s results could be an anomaly; illustrative of differences in group demographics (Martin et al.’s participants were not depressed), or indicate that the young women in the original study were already highly motivated to begin contraception use.

#### 3.3.2. Unplanned analyses

To examine whether participants in the HS group’s perceived utility or perceived resources influenced success of the MCII intervention, a mixed ANOVA utilized the questions inspired by Fleig et al. (2017). Despite Study 2 boasting a larger sample size of individuals in the HS group (*n* = 95 ITT and *n* = 75 PP) the perceived utility of setting a goal and perceived resources to achieve the goal did not appear to significantly influence the success of the intervention over time. Considering the means for the perceived resources and perceived utilities at both T1 and T2 were all greater than five (“Agree”) out of a possible score of seven for each item for both the PP and ITT analyses, it is arguable that a ceiling effect occurred. Although not mentioned in text, this was also examined using PROCCESS mediation analyses to examine whether utility and resources mediated the relationship between SIIS HS and HS MCII success as a continuous variable. Regardless of running with T1 or T2 SIIS (ITT and PP), none of the indirect effects in the analyses were significant. Further, when focusing only the regression analyses for utility, resources, and interactions of utility and resources on HS success, all analyses were insignificant.

The results of Exploratory Analyses 2a and 2b that individuals in the HS group who did not perceive themselves as depressed at T2 or whose BDI-II scores fell below the mild threshold for depression were proportionally *more likely* to report success at accomplishing their help-seeking goal were interesting findings. Conversely, perceived and actual depression scores were *not predictive of reported success* for individuals randomly assigned to the C group.

As a reminder, all participants entered the study with at least a score of 14 on the BDI-II indicating a mild level of depressive symptomatology (Hautzinger et al., 2009) at T1, 2 weeks prior to the success being measured at T2. For the individuals whose BDI-II scores at T2 indicated they no longer met a minimal threshold of depression (scores less than 14) and that they were successful at seeking help, it is impossible to establish order effects. It is just as possible that their episode of depression decreased to the point that seeking help was perceived as manageable *or* that because of seeking help, their scores on the BDI-II decreased. Using an ecological momentary assessment (EMA: Shiffman et al., 2008; Wenze and Miller, 2010) as a follow-up that can consistently measure depressive symptomatology and help-seeking actions similar to Kenny et al. (2016) could provide a clearer picture of causal relationships.

As far as perceived depression–one’s *felt* depression *regardless of their objective level of depression*– the observed results are more curious. Of the 25 individuals in the HS group who perceived themselves as non-depressed and successful at seeking help at T2, 16 had scores on the BDI-II at T2 indicating at least a mild level of depressive symptomatology. Of those 16 individuals, half did not perceive themselves as depressed at T1. A larger sample should be used to further elucidate the relationships between actual and perceived depression and any potential influence it may have on help-seeking outcomes.

It may be that an individual’s perception of “being depressed” could influence the effects of cognitive bias. Perhaps the individuals who did not perceive themselves as depressed despite meeting the mild criteria were comparing their current symptoms to others they have known who have experienced depression (e.g., social comparison theory of Festinger, 1954). An alternative is since the BDI-II inquires about depression symptoms during the previous 2 weeks (Hautzinger et al., 2009) and success did not inquire about *when* participants sought help during the intervention period, their symptoms of depression could have been subsiding for some time. Additionally, it is possible that the sample included individuals who have been diagnosed with other mental health disorders that include depressive symptoms (e.g., bipolar), potentially limiting their self-perception of “having depression” and providing a potential area for future studies.

#### 3.3.3. Summary

With the modifications from Study 1, a larger sample was obtained and retained despite an early termination due to concerns about how the emerging COVID-19 pandemic could affect the results in March 2020. Over the past few years, the pandemic and the effects of quarantining have placed mental health awareness in the spotlight due to the observed rising depression rates (see Ettman et al., 2020; Hyland et al., 2020). The inability to access care face-to-face increased the necessity to reach out to others in different ways due to social distancing. Many individuals face new life stressors such as illness or death in their families or loss of income (Nelson et al., 2020). It is difficult to predict how the intervention results of this study may have varied if the data were collected 6 or 9 months later, but it is highly likely depression ratings would be greater (see Ettman et al., 2020; Hyland et al., 2020). The general discussion includes contributions to literature, limitations, and suggestions for future studies.

## 4. General discussion

Few would deny that depression is a serious condition, and emerging evidence indicates that the rates of depression (along with other mental health issues) has risen exponentially during the COVID-19 pandemic (Cao et al., 2020; Ettman et al., 2020; Vindegaard and Benros, 2020; Wang M. et al., 2021). Although Ettman et al. (2020) noted that increasing rates of mental health concerns is common during times of uncertainty and disruption such as after the attacks on the World Trade Towers and stock market crashes, the novel coronavirus may provide unique and lasting challenges for a wider segment of the population. Dubey et al.’s (2020) review of the literature found that factors such as forced quarantines, job insecurities, pervasive negative feelings (including guilt, inadequacy, or fear), scarcity of basic resources, on top of fear of the illness contributed to reduced feelings of wellbeing among individuals with little regard for age, gender, or occupation. In addition to confirming the role of the psychosocial factors listed above leading to increased depression, Wang M. et al. (2021) noted that for individuals with preexisting conditions, there is increased stress due to the inability to schedule or attend appointments. For these reasons, a simple online intervention that can encourage help-seeking for depression from a multitude of sources seems particularly timely.

Despite random assignment to conditions and technically reaching the target number of participants required by the power analysis after attrition and data cleaning in Study 1 (g*power estimate *n* = 72, achieved PP *n* = 74), the control group (C *n* = 40) was still twice as large as the experimental (HS *n* = 17) and comparison groups (E *n* = 17). It was surprising that even with the small number of participants that completed the HS MCII, there was a statistically significant difference such that those in the HS group were more likely to report an increase in their implementation intentions to seek-help (as measured by the SIIS HS) than those in the E or C groups regardless of their actual help-seeking behaviors for those in the PP sample. Study 1 established that although modifications were necessary, the online HS MCII intervention for use with MTurk populations was feasible.

With modifications to the length of survey and over-sampling, attrition was reduced from ~40% in Study 1 to ~25% in Study 2. The significant intervention results in H1 and H2a appeared more promising in Study 2. By adding the depression demographic measures to T2, it was possible to perform the supplementary analyses. However, interpreting the results of those analyses requires caution since the individuals who were proportionally the most likely to be successful were in the HS group and did not *perceive* themselves as having elevated depressive symptomatology or their depressive symptomatology decreased from T1 to T2. An alternative hypothesis proposed by Nisbett and Wilson (1977) is that individuals may not have cognitive access or awareness of their depression status and therefore, any reports of their depression status may be suspect (see also Johansson et al., 2005; Petitmengin et al., 2013). Without further exploration with a larger sample and using a technique such as EMA (Shiffman et al., 2008; Wenze and Miller, 2010), it would be impossible to establish a timeline of changes in depression scores (or changes in negative bias) and individual help-seeking actions (see Kenny et al., 2016).

### 4.1. Limitations and strengths

Although samples collected via MTurk’s TurkPrime, which is based on self-selection, offers more diversity than an average college class, as noted by Casler et al. (2013), samples consistently more likely to be white (see also McCredie and Morey, 2019) and suffer from high attrition rates (Zhou and Fishbach, 2016). However, it is also possible that the demographics were homogeneous because of limiting the sample to US residents who were proficient in English. McCredie and Morey (2019) found that MTurk participants may vary from community samples by being more socially isolated, having a more limited social support network, and having higher depression scores (p. 764). Although their findings suggest limitations for some topics, these characteristics suggest that MTurk samples may provide a fertile ground to test the present intervention. Although the initial MTurk HIIT did not advertise that this study was specifically related to help-seeking for depression, no deception was used in the consent form indicating that there was self-selection into this study since all individuals could opt out at any point. This was also reflected by rates of self-reporting at least mild levels of depressive symptomatology recruited for both Studies (Study 1: 32.4%; Study 2: 33.8%) that are a little higher than recently reported statistics on pre-pandemic depressive symptomatology (pre-pandedemic: 24.7%, March 2020–June 2021: 36%; See Ettman et al., 2020, 2023). It should also be noted that the BDI-II is a depression screening measure, but it does not take the place of a clinical diagnosis of depression. Relevance of the intervention to the MTurk population could also help explain why the current studies collected slightly larger sample sizes than previous MCII mental health studies (e.g., *N* = 47, Fritzsche et al., 2016; *N* = 36, Sailer et al., 2015).

The rationale for using ITT analyses is to reduce bias when conducting randomized control trials. As was noted, rather than using a traditional ITT method where all participants who were randomized are analyzed (see Day, 2008; Gupta, 2011), this study preestablished the necessity of passing the quantitative attention checks. This modification seemed pertinent to add as a layer of protection against fraudulent data often used with MTurk samples (e.g., Kennedy et al., 2020). A key challenge for the current studies is that with only two time points and significant attrition rates, making plausible assumptions to impute the missing data (e.g., using the carry forward method, multiple imputation) would also create undo bias. White et al. (2011) offers several suggestions to combat these issues including vigilant follow ups and reducing attrition using study design; both methods these studies employed. To assess potential bias in attrition rates in the current studies, the modified ITT samples for each study was compared by condition (i.e., Study 1: SH, E, C; Study 2: SH, C) and time point (i.e., T1, T2) and no statistically significant differences were found. To be clear, there are many ways to approach ITT analyses, each with their own caveats; it is possible using another method might have returned different results.

There are many methods that can be used to form and reinforce implementation intentions, ranging from simply reading a preformed implementation intention (e.g., Gawrilow and Gollwitzer, 2007; Parks-Stamm et al., 2010) to the more in-depth MCII interventions (e.g., Sailer et al., 2015; Fritzsche et al., 2016) including the specific WOOP strategy (e.g., Mutter et al., 2020; Monin et al., 2021). Other studies have successfully used tools such as volitional help sheets whereby the participants were aided in forming implementation intentions for multiple situations related to the topic (e.g., Armitage, 2015; Armitage et al., 2016). Given the many ways implementation intentions can be formed and reinforced, one limitation of this set of studies is the sole utilization of the online MCII. The decision to do so was based on the success of MCII with individuals with elevated depressive symptomatology (e.g., Sailer et al., 2015; Fritzsche et al., 2016) but future studies may consider exploring if this approach is best by comparing it with other techniques for establishing implementation intentions. For example, a volitional help sheet that outlines multiple help-seeking options may be just as–or more–useful for this population.

All measures used in this study and the measure of success was dependent on self-report; a possible limitation. This procedure is not unusual in the field of MCII (Webb and Sheeran, 2006) or depression research (Keeler and Siegel, 2016), but should be noted. Although it is certainly more resource efficient (i.e., time, money), it is possible that individuals will not be honest or will forget about their help-seeking practices. However, while self-report is less than perfect, the added opportunity to reiterate the MCII in the form of a quantitative scale, directions to keep a copy of the help-seeking information, and the personalized implementation on top of the relatively short time period may reduce memory errors across groups. In general, few individuals were removed due to completely forgetting their implementation intention. It should also be noted that apart from asking two questions in Study 2 regarding whether participants sought help from interpersonal or professional sources, we did not require participants to qualify what help-seeking behaviors they initiated in the past 2 weeks; only if they were successful at initiating their personalized help-seeking plan.

Studying the HS MCII intervention effects add significantly to the literature in several ways. As mentioned, although the literature using implementation intentions and mental health has grown substantially since Gollwitzer and Sheeran’s (2006) meta-analysis, interventions directed specifically at individuals with depression are quite rare (e.g., Fritzsche et al., 2016). Quite often, individuals with depression may be included in studies (e.g., Sheeran et al., 2007; Pomp et al., 2013; Armitage et al., 2016) or depression may be considered as a variable (e.g., Sailer et al., 2015; Mutter et al., 2020) but it is not the exclusive focus. Given the conflicting results of the previous studies that measured depression explicitly (i.e., successful: Fritzsche et al., 2016; unsuccessful: Pomp et al., 2013), this study provided much needed support for the viability of using implementation intentions among individuals with elevated depressive symptomatology. This study may serve as a step in supporting the premise of how the process of completing an MCII can limit the effects of negative bias to encourage achieving a goal beyond receiving information alone. This appeared to happen with those in the HS MCII condition.

Although the results should be replicated to fully consider the differences in reporting timeframes of the BDI-II, perceived depression, and goal achievement, the most significant addition to the literature is the potential of adding MCII as another tool to initiate help-seeking for depression. As mentioned, the current mental health implementation intention literature is nearly devoid of help-seeking interventions. The only implementation intention study located focused specifically on helping individuals *follow through* with a previously scheduled appointment with a mental health professional (i.e., Sheeran et al., 2007). A recent literature search in mid 2022 failed to find any studies that expressly examined MCII for depression using MTurk. Though an MCII intervention was successful at initiating help-seeking for individuals to establish behaviors to help overcome chronic back pain (Christiansen et al., 2010), the use for the initiation of mental health support has not been previously explored. Given the number of interventions designed to encourage help-seeking that have had iatrogenic effects (e.g., Lienemann et al., 2013; Keeler and Siegel, 2016), it is important to find innovative and reliable ways to encourage formal and informal help-seeking in relatively simple to disseminate that is easy to tailor to individuals. Such research could make a practical and important impact considering escalating depression rates due to the COVID-19 pandemic (Cao et al., 2020; Ettman et al., 2020; Vindegaard and Benros, 2020; Wang M. et al., 2021).

### 4.2. Future directions

Above all, we suggest replication and expansion studies that address the limitations noted with larger, more diverse samples and via different recruitment modalities (i.e., cloud research, community, health clinic). With further research, it will be possible to delineate exactly how this method can be applied in the future to examine possible dissemination to a wider audience to encourage various help-seeking initiation behaviors like other mental health interventions including through mass media (e.g., Ort and Fahr, 2022), clinics (e.g., Monin et al., 2021), or workplace initiatives (e.g., Gollwitzer et al., 2018). Thus, more research is needed to replicate findings and expand reach.

As a first step, future studies should seek to both generalize to symptomatology beyond depression as well as seek to explore combinations of symptom severity. It is currently unknown if a mental health help-seeking MCII intervention would be effective for individuals who may not be actively experiencing negative bias (e.g., anxiety, mania).

Considering the results regarding the differences in BDI-II scores in the exploratory analyses, future studies may want to explore the nuance between the severity of disorders and the type of implementation intention interventions that work best at each level for encouraging help-seeking. Currently, assessing the level of severity of mental health disorders has been largely unexplored with the exception of Parks-Stamm et al. (2010), who examined the effectiveness of the type of implementation intention intervention most useful for shielding individuals with varying levels of test anxiety from unwanted distraction. Their results indicated it is vital to explore severity of conditions because what is useful at a mild level of impairment may not be effective at a more severe level. In the case of depression, a simpler implementation intention (“If I am feeling depressed, I will call my loved one”) may be sufficient to increase help-seeking for depression for someone with mild symptoms, whereas the more complex MCII interventions (i.e., elaborating on the positive and negative aspects of calling a loved one for help) with multiple follow ups or in-person training may be needed for moderate to severe levels of depression. This knowledge would be useful in that resources (e.g., time, money for training) can be more efficiently directed to the populations that need it the most.

Galea et al.’s (2020) recent call for innovative ways to increase training for non-traditional mental health first aid suggests an alternative population interest for future MCII aimed to care for individuals with elevated depressive symptomatology. HS MCII interventions could potentially be utilized for the family member to recognize when they should intervene with their loved one with depression and have pre-established responses to best offer support to their loved ones (e.g., “If my loved one talks about suicide, then I will call the crisis intervention hotline saved on my phone”). Due to the high recurrence rate of depression (Judd et al., 2000), it may be interesting and useful to design an MCII intervention for the loved ones of individuals with mental health symptoms as part of an aftercare plan. For example, the loved ones could form specific implementation intentions for what, when, and how they will provide their loved one with help if they see the symptoms of depression reoccur (“If I notice my loved one stops going to their aftercare treatment, then I will offer to drive them to their next appointment”). Although there is limited evidence in this area, a recent study found that focusing on caregivers can benefit both the caregiver and their loved one’s mental health (Monin et al., 2021).

Although the current intervention seeks to explore if MCII techniques can help initiate help-seeking for depression over a 2 week time period, it would be interesting to test if combining MCII techniques with aspects of a complementary model such as Siegel et al. (2010) IIFF model could bolster intervention effectiveness. The IIFF Model, designed to increase organ donation registration, advocates for increasing information, favorable activation of the desired behavior, focused engagement, and an immediate and complete opportunity to engage in the behavior. A joint MCII and IIFF help-seeking for depression intervention would include providing information that is specifically favorable to the multiple avenues of help-seeking, which is already standard in many help-seeking campaigns. Due to mental contrasting’s proposed tempering of negative bias due to the more balanced approach to deciding to seek help and acknowledging the negative reality, a favorable view of help-seeking may not have the same boomerang effect previous studies have found (e.g., Keeler and Siegel, 2016). Mental contrasting and implementation intentions are designed to provide focused engagement with an issue by having the individual actively contemplate not only their positive and negative realities of initiating help-seeking but also setting a plan.

Where IIFF could add to MCII is by answering the question of whether providing an *immediate* opportunity for help-seeking would be efficacious. This could be accomplished by using an online study and providing participants with depression the opportunity to go directly to a link for a national crisis center information page, directly link to the suicide lifeline chat service, connect to a professional, or to open a window to send an email to a loved one for help.

### 4.3. Conclusion

At a time when depression rates are increasing because of the lingering COVID-19 pandemic (Cao et al., 2020; Ettman et al., 2020; Vindegaard and Benros, 2020; Wang M. et al., 2021), it is vital to develop remote, affordable, scalable, and effective interventions to encourage help-seeking. Together, this set of studies offers support that a brief online MCII intervention to increase help-seeking initiation and intentions to seek help is feasible and offers preliminary evidence of success. However, whether actual help-seeking initiation success is based *solely* on the intervention requires further investigation. Future studies should address the limitations of this study and consider using EMA measurements to establish temporal precedence regarding the finding that those who were more likely to report successful help-seeking reported decreased BDI-II scores at T2. Examining larger samples of participants with varying severities and differing mental health symptoms could also provide insight into the effectiveness of MCII for encouraging mental health help-seeking among individuals prone to experiencing cognitive errors who may not be experiencing negative bias (e.g., bipolar disorder or anxiety). Clinicians may find this method successful for encouraging continued attendance to treatment sessions as well as targeting loved ones to recognize warning signs and plan a strategy for intervening with a loved one with depression.

## Data availability statement

The raw data supporting the conclusions of this article will be made available by the authors, without undue reservation.

## Ethics statement

The studies involving human participants were reviewed and approved by the Claremont Graduate University. Written informed consent for participation was not required for this study in accordance with the national legislation and the institutional requirements.

## Author contributions

AK, LN, and WC contributed to the conception and design of this manuscript. LN and AK modified the key measurement. AK created the database, performed the statistical analyses, and wrote the first draft of the manuscript. WC provided the statistical support. LN completed tables and figures. All authors contributed to the manuscript revision, read, and approved the submitted version.

## Funding

This publication was supported by the Health Resources and Services Administration (HRSA) of the U.S. Department of Health and Human Services (HHS) as part of an award totaling $590,270.00 with 14% financed with non-governmental sources. The contents are those of the author(s) and do not necessarily represent the official views of, nor an endorsement, by HRSA, HHS, or the U.S. Government. For more information, please visit HRSA.gov.

## Conflict of interest

The authors declare that the research was conducted in the absence of any commercial or financial relationships that could be construed as a potential conflict of interest.

## Publisher’s note

All claims expressed in this article are solely those of the authors and do not necessarily represent those of their affiliated organizations, or those of the publisher, the editors and the reviewers. Any product that may be evaluated in this article, or claim that may be made by its manufacturer, is not guaranteed or endorsed by the publisher.

## Footnotes

^1^Although the power analysis indicated that a sample size of 72 was required and the total sample sized met this goal, the small and unequal group sizes may have resulted in inadequate power.

^2^After an additional reminder to complete the final survey, the T2 surveys were deactivated on 3/18/20 due to the growing COVID-19 pandemic since the effects of the worsening global situation might be impossible to disentangle from the intervention effect.

## Abbreviations

BDI-II: Beck Depression Inventory-second edition
C: Information-Only Control
CTD: Cognitive theory of depression
E: Exercise
EMA: Ecological momentary assessment
HS: Help-seeking
ITT: Intention to Treat
MCII: Mental contrasting and implementation intentions
PP: Per Protocol
SIIS: Strength of Implementation Intentions Scale
T1: Time 1
T2: Time 2
