# Supplementary material for: Combatting negative bias: a mental contrasting and implementation intentions online intervention to increase help-seeking among individuals with elevated depressive symptomatology
Source: Front Psychol. 2023 Jun 15;14:1145969. doi: 10.3389/fpsyg.2023.1145969 (PMC10310967; doi:10.3389/fpsyg.2023.1145969)
Supplement: Supplementary file 1 [file Table_1.DOCX]

Supplementary Material

Combatting negative bias: A mental contrasting and implementation intentions online intervention to increase help-seeking among individuals with elevated depressive symptomatology

Amanda R Keeler^1*^, Liesl A Nydegger^2^, William D Crano^3^

*** Correspondence:** Corresponding Author: [mandakeeler@me.com](mailto:mandakeeler@me.com)

# Supplementary Help-Seeking Information

Many people go through periods of time when they feel distressed or hopeless. This could be

when they are going through difficult transitions or while processing complex feelings. It’s common to feel like there is nowhere to turn. However, **individuals experiencing depression are not alone. There are many places where people experiencing those feelings can turn to feel better**.

For example, individuals can talk to their family and friends about how they are feeling. For those who feel more comfortable talking to someone outside of their family and friends, there are many free or low-cost options for support and guidance.

One option available for individuals who would like to talk to someone when distressed is the National Suicide Prevention Lifeline. The lifeline can be contacted at any time by calling the 24/7 toll-free number 1-800-273-TALK (8255) or by visiting [www.suicidepreventionlifeline.org.](http://www.suicidepreventionlifeline.org/) For individuals who feel more comfortable texting, crisis counselors can be reached 24/7 in the United States by texting HOME to 741741 (standard messaging rates may apply) or by visiting their website https://[www.crisistextline.org.](http://www.crisistextline.org/) These are among just a few of the resources that are readily available for individuals who are experiencing distress. **Individuals with depression never have to be alone in their struggle – there are places and services that can help**.

Please save this information in case someone you or someone you know ever experiences depression and needs someone to talk to.
